# Synthetic photonic lattices based on three-level giant-atom arrays

**DOI:** 10.1016/j.fmre.2024.03.029

**Published:** 2024-05-09

**Authors:** Lei Du, Yan Zhang, Xin Wang, Yong Li, Yu-xi Liu

**Affiliations:** a Center for Theoretical Physics & School of Physics and Optoelectronic Engineering, Hainan University, Haikou 570228, China; b Department of Microtechnology and Nanoscience, Chalmers University of Technology, Gothenburg 41296, Sweden; c School of Physics and Center for Quantum Sciences, Northeast Normal University, Changchun 130024, China; d Institute of Theoretical Physics, School of Physics, Xi’an Jiaotong University, Xi’an 710049, China; e School of Integrated Circuits, Tsinghua University, Beijing 100084, China

**Keywords:** Giant atom, Synthetic photonic lattice, Circuit QED, Decoherence-free interaction, Quantum network engineering

## Abstract

Simulating photonic lattices remains to be an interesting and important goal for quantum technologies. Here, we propose several simulation schemes of one- and quasi-one-dimensional photonic lattices based on arrays of diverse three-level giant-atom dimers. The resulting models, including diamond, Su-Schrieffer-Heeger, and ladder lattices, exhibit protected nearest-neighbor and greatly inhibited next-nearest-neighbor interactions, which are challenging with most state-of-the-art experimental platforms. Our proposals based on circuit quantum electrodynamics are tunable, scalable, and reconfigurable, thus providing opportunities for simulating more advanced photonic lattices and exploring unprecedented phenomena with no counterparts in conventional condensed matter physics.

## Highlights

• It is the first time the decoherence-free interactions between three-level giant atoms are systematically studied.
• We propose feasible simulation schemes for several prototypical photonic lattice models, which are highly protected from decoherence and can be programmed in situ.
• Our proposals can be readily implemented in a lab with state-of-the-art circuit QED techniques and can outperform other conventional schemes in terms of many figures of merit.

## Graphical abstract

**Figure:**
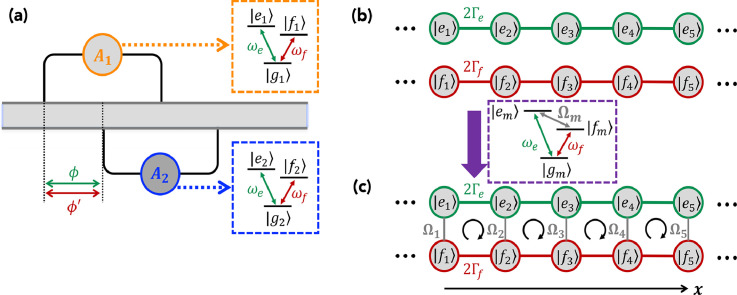

## 1 Introduction

Simulating the evolution of quantum particles in various periodic structures is a crucial and long-standing task in quantum mechanics, with potential applications in fields ranging from quantum many-body physics to quantum information processing. It is believed that photons are excellent quantum information carriers with low noise, long coherence length, and high transmission speed [1]. Therefore, photonic lattices provide a promising platform for engineering light-matter interactions and large-scale quantum networks, allowing for phenomena absent in conventional condensed matter physics. The research interest in photonic lattices is rising rapidly, with the implementation candidates including coupled waveguide arrays [2], [3], photonic crystals [4], superconducting quantum circuits [5], [6], and optomechanical systems [7], [8], [9], to name a few. Moreover, recent progress shows that it is possible to create lattice structures in synthetic dimensions, such as those based on the frequency [10], [11], [12], momentum [13], orbital angular momentum [14], [15], [16], and Fock states of photons [17]. More interestingly, it was recently shown that photonic lattices can be used to simulate curved spaces, both in real space and through a specific mapping [18], [19]. These exciting breakthroughs not only find applications in computational science but also show the possibility towards unifying quantum mechanics and general relativity.

In this paper, we study a new quantum optical paradigm that allows for simulating photonic lattices with *tunable architectures and protected interactions*. The building block here is the so-called “giant atom” [20], which is coupled to a guided field at two or more separate points and can be readily implemented in experiments. Such a device can behave as a sort of tiny quantum interferometer, which exhibits self-interference effects that depend on the phase accumulations of the field traveling between different coupling points [21], [22], [23], [24], [25], [26]. An important hallmark of giant atoms is their ability to interact with each other in a decoherence-free manner, i.e., the atoms exchange energy without relaxing into the waveguide field, even if the atomic frequency is within an energy band of the waveguide [27], [28]. In contrast to conventional schemes where decoherence-free interactions between normal atoms (i.e., atoms that are locally coupled to the waveguide) are realized through virtual photon processes mediated by localized photon-atom bound states [29], [30], in the giant-atom case the interactions can have very long range, and one does not have to engineer band gaps for the waveguide to create photon-atom bound states.

The in-band decoherence-free interactions between *two-level* giant atoms have been demonstrated to be a powerful tool for simulating one-dimensional (1D) tight-binding chains with protected and tunable nearest-neighbor couplings [27]. These couplings can be complex (i.e., mimicking synthetic magnetic flux) by properly modulating the atom-field interactions or the atomic transition frequencies [31]. Moreover, it is also feasible to simulate the Su-Schrieffer-Heeger (SSH) model [32], [33], which is the simplest (Hermitian) topological lattice model, with two-level giant atoms based on either chiral photon-atom bound states [34] or staggered decoherence-free interactions [35]. Considering that atoms with more energy levels involved can exhibit richer quantum interference effects and more control parameters, it is natural to expect more advanced lattice structures by choosing appropriate *multi-level* giant atoms. Motivated by this, we here study several atomic dimer models based on different combinations of three-level Λ- and V-type giant atoms and demonstrate how to simulate diverse photonic lattices by extending them to well-designed arrays. Our proposals show a series of advantages compared with other conventional ones and are highly relevant to state-of-the-art experimental platforms such as superconducting quantum circuits.

The rest of this paper is organized as follows. In Section 2.1, we study an atomic dimer model consisting of a Λ- and a V-type giant atoms and then demonstrate how to simulate a diamond lattice with an array of such dimers. In Section 2.2, we study a double-Λ model and simulate the SSH model based on the extended array. In Section 2.3, we study a double-V model and construct a synthetic ladder lattice by introducing external driving fields to the extended array. In Section 3, we discuss the experimental implementations and the advantages of our proposals and conclude our work. In the supplementary material, we provide the derivation procedure of the time-delayed dynamical equations of the Λ−V model in Section 2.1 and the analytical expressions of the eigenvalues of a more general synthetic diamond lattice, possibly with middle band gaps.

## 2 Results and discussion

### 2.1 Λ−V model

**Fig. 1:**
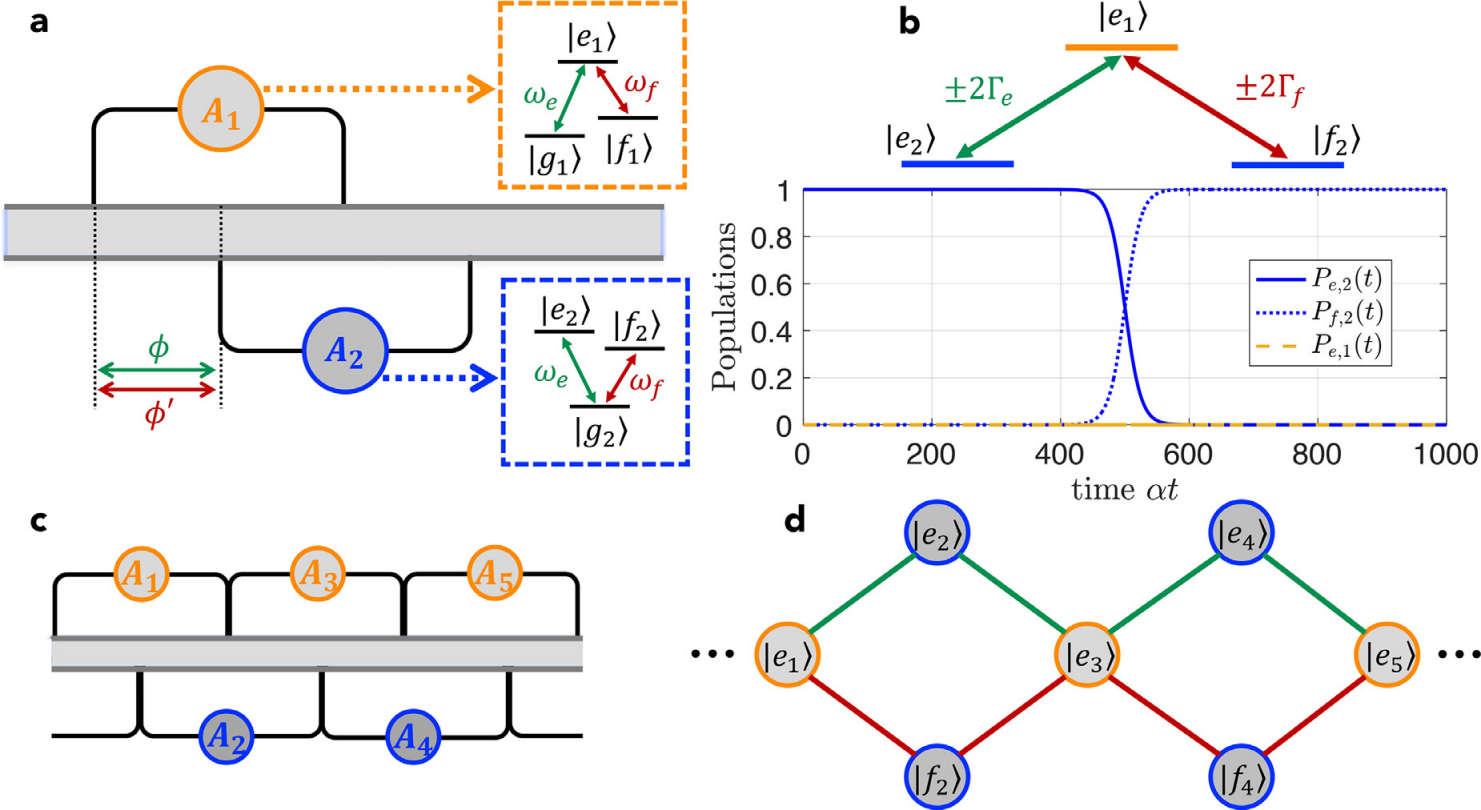
**(a) Schematic diagram of the**Λ−V**model**. The Λ- and V-type three-level giant atoms are coupled to a common 1D waveguide with an equally spaced braided structure. (b) Effective Λ-type energy-level configuration under the decoherence-free condition (upper) and the corresponding STIRAP with $n=n^{\prime }=1$, $\alpha t_{1}=400$, $\alpha t_{2}=600$, and αT=100 (lower). (c) Λ−V model array with only nearest-neighbor decoherence-free interactions. (d) Effective diamond lattice based on the array in panel (c).

We first consider a pair of giant atoms with Λ- and V-type three-level structures, respectively, as schematically shown in Fig. 1a. The two atoms are coupled to the waveguide in a braided structure [27], [28], with each having two identical coupling points. For simplicity, we assume that the coupling points are equally spaced by distance d, but this is not the essential condition of the results in this paper (decoherence-free interactions between giant atoms can be realized even if the braided coupling points are not equally spaced [27], [28]). The Λ-type atom (labeled as $A_{1}$) has one upper state $|e_{1}\rangle$ and two lower states $|f_{1}\rangle$ and $|g_{1}\rangle$ (with frequencies $\omega_{e,1}$, $\omega_{f,1}$, and $\omega_{g,1}$, respectively), while the V-type atom (labeled as $A_{2}$) has two upper states $|e_{2}\rangle$ and $|f_{2}\rangle$ and one lower state $|g_{2}\rangle$ (with frequencies $\omega_{e,2}$, $\omega_{f,2}$, and $\omega_{g,2}$, respectively). Moreover, the energy-level transitions of the two atoms are well designed so that $\omega_{e,1}-\omega_{g,1}=\omega_{e,2}-\omega_{g,2}\equiv \omega_{e}$ and $\omega_{e,1}-\omega_{f,1}=\omega_{f,2}-\omega_{g,2}\equiv \omega_{f}$ (this condition can be appropriately relaxed as will be shown below). In experiments, such a model can be readily achieved with superconducting quantum circuit platforms, as discussed in detail in Section 3. Based on the above assumptions, the Hamiltonian of this braided Λ−V model can be written as (ℏ=1 in this paper)

(1) $$H=H_{\text{w}}+H_{A_{1}}+H_{A_{2}}+V_{\text{w}1}+V_{\text{w}2},$$

(2) $$H_{\text{w}}=\int_{-\infty }^{+\infty }dk\omega_{k}a_{k}^{†}a_{k},$$

(3) $$H_{A_{1}}=\omega_{e}|e_{1}\rangle \langle e_{1}|+\Delta_{ef}|f_{1}\rangle \langle f_{1}|,$$

(4) $$H_{A_{2}}=\omega_{e}|e_{2}\rangle \langle e_{2}|+\omega_{f}|f_{2}\rangle \langle f_{2}|,$$

(5) $$V_{\text{w}1}=\int_{-\infty }^{+\infty }dk\left(1+e^{2ikd}\right)\left(g_{e}|e_{1}\rangle \langle g_{1}|+\;g_{f}|e_{1}\rangle \langle f_{1}|\right)a_{k}+\text{H.c.},$$

(6) $$V_{\text{w}2}=\int_{-\infty }^{+\infty }dk\left(e^{ikd}+e^{3ikd}\right)\left(g_{e}|e_{2}\rangle \langle g_{2}|+g_{f}|f_{2}\rangle \langle g_{2}|\right)a_{k}+\text{H.c.},$$

where $a_{k}$ ($a_{k}^{†}$) is the annihilation (creation) operator of the waveguide mode with wave vector k and frequency $\omega_{k}=v_{g}\left|k\right|$ ($v_{g}$ is the group velocity of the waveguide modes); $g_{e}$ and $g_{f}$ are the coupling coefficients between the waveguide field and the atomic transitions with frequencies $\omega_{e}$ and $\omega_{f}$, respectively, which are assumed to be real constants under the Weisskopf-Wigner approximation and identical for the two atoms; $\Delta_{ef}=\omega_{e}-\omega_{f}$ is the difference between the two atomic transition frequencies; and the positions of the four coupling points are assumed as $\left\{x_{1},x_{2},x_{3},x_{4}\right\}=\left\{0,d,2d,3d\right\}$ without loss of generality. Considering that the total excitation number of the model is conserved, i.e., $[\hat{N},H]=0$ with $\hat{N}=\int dka_{k}^{†}a_{k}+|e_{1}\rangle \langle e_{1}\left|+\right|e_{2}\rangle \langle e_{2}\left|+\right|f_{2}\rangle \langle f_{2}|$ the operator of the total excitation number, and assuming that $A_{1}$ ($A_{2}$) is initially prepared in state $|e_{1}\rangle$ ($|g_{2}\rangle$), one can study the dynamics of the model in the single-excitation subspace and write the state at time t as

(7) $$\begin{array}{ccc} |\psi (t)\rangle & = & \left[c_{e,1}\left(t\right)|e_{1}g_{2}\rangle +c_{e,2}\left(t\right)|g_{1}e_{2}\rangle +c_{f,2}\left(t\right)|f_{1}f_{2}\rangle \right]e^{-i\omega_{e}t}|0\rangle \\ & & +\;\int_{-\infty }^{+\infty }dkc_{k}\left(t\right)a_{k}^{†}e^{-i\omega_{k}t}|g_{1}g_{2}\rangle |0\rangle \\ & & +\;\int_{-\infty }^{+\infty }dkc_{k}^{\prime }\left(t\right)a_{k}^{†}e^{-i\Delta_{ef}t}e^{-i\omega_{k}t}|f_{1}g_{2}\rangle |0\rangle , \end{array}$$

where $c_{e,1}$, $c_{e,2}$, and $c_{f,2}$ are the time-dependent probability amplitudes with the waveguide in the vacuum state |0〉 and the atomic states $|e_{1}\rangle$, $|e_{2}\rangle$, and $|f_{2}\rangle$ being populated, respectively; $c_{k}$ ($c_{k}^{\prime }$) is the time-dependent probability amplitude of creating a photon in the waveguide and atoms $A_{1}$ and $A_{2}$ in states $|g_{1}\rangle$ ($|f_{1}\rangle$) and $|g_{2}\rangle$, respectively. Here we have assumed that $\Delta_{ef}$ is large enough ($|\Delta_{ef}\left|\gg \{\right|g_{e}\left|,\right|g_{f}\left|\right\}$) such that a photon with frequency $\omega_{e}$ ($\omega_{f}$) can hardly be coupled to the atomic transition of frequency $\omega_{f}$ ($\omega_{e}$). By solving the time-dependent Schrödinger equation $i\partial_{t}|\psi \left(t\right)\rangle =H|\psi \left(t\right)\rangle$, one can obtain the dynamical equations:

(8) $$i{\dot{c}}_{e,1}\left(t\right)=\int dk\left(1+e^{2ikd}\right)\left[g_{e}c_{k}\left(t\right)e^{-i\Delta_{e}t}+g_{f}c_{k}^{\prime }\left(t\right)e^{-i\Delta_{f}t}\right],$$

(9) $$i{\dot{c}}_{e,2}\left(t\right)=\int dkg_{e}\left(e^{ikd}+e^{3ikd}\right)c_{k}\left(t\right)e^{-i\Delta_{e}t},$$

(10) $$i{\dot{c}}_{f,2}\left(t\right)=\int dkg_{f}\left(e^{ikd}+e^{3ikd}\right)c_{k}^{\prime }\left(t\right)e^{-i\Delta_{f}t}$$

for the atomic excitation amplitudes and

(11) $$i{\dot{c}}_{k}\left(t\right)=g_{e}\left[\left(1+e^{-2ikd}\right)c_{e,1}\left(t\right)+\left(e^{-ikd}+e^{-3ikd}\right)c_{e,2}\left(t\right)\right]e^{i\Delta_{e}t},$$

(12) $$i{\dot{c}}_{k}^{\prime }\left(t\right)=g_{f}\left[\left(1+e^{-2ikd}\right)c_{e,1}\left(t\right)+\left(e^{-ikd}+e^{-3ikd}\right)c_{f,2}\left(t\right)\right]e^{i\Delta_{f}t}$$

for the field excitation amplitudes, where $\Delta_{e}=\omega_{k}-\omega_{e}$ and $\Delta_{f}=\omega_{k}-\omega_{f}$ are the detunings between waveguide mode $a_{k}$ and the two atomic transitions. Assuming that the waveguide is in the vacuum state initially, it is straightforward to write down the formal solutions of $c_{k}\left(t\right)$ and $c_{k}^{\prime }\left(t\right)$, i.e.,

(13) $$c_{k}\left(t\right)=-i\int_{0}^{t}dt^{\prime }g_{e}\left[\left(1+e^{-2ikd}\right)c_{e,1}\left(t^{\prime }\right)+\left(e^{-ikd}+e^{-3ikd}\right)c_{e,2}\left(t^{\prime }\right)\right]e^{i\Delta_{e}t^{\prime }},$$

(14) $$c_{k}^{\prime }\left(t\right)=-i\int_{0}^{t}dt^{\prime }g_{f}\left[\left(1+e^{-2ikd}\right)c_{e,1}\left(t^{\prime }\right)+\left(e^{-ikd}+e^{-3ikd}\right)c_{f,2}\left(t^{\prime }\right)\right]e^{i\Delta_{f}t^{\prime }}.$$

By substituting Eqs. 13 and 14 into (8), (9), (10) and following a standard derivation procedure (for more details see Sec. I in the supplemental material), the time-delayed dynamical equations of the atomic excitation amplitudes can be obtained as

(15) $$\begin{array}{ccc} {\dot{c}}_{e,1}\left(t\right) & = & -\left(\Gamma_{e}+\Gamma_{f}\right)c_{e,1}\left(t\right)-\left(\Gamma_{e}e^{2i\phi }+\Gamma_{f}e^{2i\phi^{\prime }}\right)D_{e,1}^{\left(2\right)}\\ & & -\;\frac{\Gamma_{e}}{2}\left[3e^{i\phi }D_{e,2}^{\left(1\right)}+e^{3i\phi }D_{e,2}^{\left(3\right)}\right]\\ & & -\;\frac{\Gamma_{f}}{2}\left[3e^{i\phi^{\prime }}D_{f,2}^{\left(1\right)}+e^{3i\phi^{\prime }}D_{f,2}^{\left(3\right)}\right], \end{array}$$

(16) $${\dot{c}}_{e,2}\left(t\right)=-\Gamma_{e}\left[c_{e,2}\left(t\right)+e^{2i\phi }D_{e,2}^{\left(2\right)}\right]-\frac{\Gamma_{e}}{2}\left[3e^{i\phi }D_{e,1}^{\left(1\right)}+e^{3i\phi }D_{e,1}^{\left(3\right)}\right],$$

(17) $${\dot{c}}_{f,2}\left(t\right)=-\Gamma_{f}\left[c_{f,2}\left(t\right)+e^{2i\phi^{\prime }}D_{f,2}^{\left(2\right)}\right]-\frac{\Gamma_{f}}{2}\left[3e^{i\phi^{\prime }}D_{e,1}^{\left(1\right)}+e^{3i\phi^{\prime }}D_{e,1}^{\left(3\right)}\right],$$

where $D_{\beta }^{\left(l\right)}=c_{\beta }\left(t-l\tau \right)\Theta \left(t-l\tau \right)$ with Θ(x) the Heaviside step function; $\phi =\omega_{e}\tau =\omega_{e}d/v_{g}$ ($\phi^{\prime }=\phi -\Delta_{ef}\tau =\omega_{f}\tau$) is the phase accumulation of a photon with frequency $\omega_{e}$ ($\omega_{f}$) traveling between two adjacent coupling points and τ is the corresponding time delay (i.e., propagation time); $\Gamma_{e}=4\pi g_{e}^{2}/v_{g}$ ($\Gamma_{f}=4\pi g_{f}^{2}/v_{g}$) is the waveguide-induced decay rate of the atomic transitions with frequency $\omega_{e}$ ($\omega_{f}$). If we consider the situation where (i) both ϕ and $\phi^{\prime }$ are *half-integer* multiples of π and (ii) the time delay τ is small enough such that $\{\Gamma_{e}\tau ,\Gamma_{f}\tau \}\ll 1$ (i.e., in the Markovian regime), we have

(18) $${\dot{c}}_{e,1}\left(t\right)=2\left[{\left(-i\right)}^{n}\Gamma_{e}c_{e,2}\left(t\right)+{\left(-i\right)}^{n^{\prime }}\Gamma_{f}c_{f,2}\left(t\right)\right],$$

(19) $${\dot{c}}_{e,2}\left(t\right)=2{\left(-i\right)}^{n}\Gamma_{e}c_{e,1}\left(t\right),$$

(20) $${\dot{c}}_{f,2}\left(t\right)=2{\left(-i\right)}^{n^{\prime }}\Gamma_{f}c_{e,1}\left(t\right)$$

with n=mod(ϕ,2π)/(π/2) and $n^{\prime }=\text{mod}\left(\phi^{\prime },2\pi \right)/\left(\pi /2\right)$ (it is clear that $\left\{n,n^{\prime }\right\}=\left\{1,3\right\}$). As shown in Fig. 1b, such a model shows a protected Λ-type configuration consisting of states $|e_{1}\rangle$, $|e_{2}\rangle$, and $|f_{2}\rangle$, with the effective Rabi frequencies determined by the atom-waveguide coupling coefficients $g_{e}$ and $g_{f}$ as well as the phase accumulations ϕ and $\phi^{\prime }$. With this effective Λ-type configuration in hand, one can implement stimulated Raman adiabatic passage (STIRAP) [36], which enables efficient and robust population transfer from $|e_{2}\rangle$ to $|f_{2}\rangle$ (or vice versa) in a coherent control manner, by properly modulating the atom-waveguide coupling coefficients $g_{e}$ and $g_{f}$ (i.e., the effective Rabi frequencies $\Gamma_{e}$ and $\Gamma_{f}$) with time. In circuit quantum electrodynamics (QED), such time-dependent coupling coefficients can be readily achieved by connecting the (artificial) atoms to the transmission line through superconducting quantum interference devices with tunable inductance and dynamically modulating their inductance via a controllable bias current [37]. In Fig. 1b we provide a proof-of-principle demonstration of STIRAP by using counter-intuitive time-dependent modulations $\Gamma_{f}\left(t\right)=\alpha \text{exp}\left[-{\left(t-t_{1}\right)}^{2}/T^{2}\right]$ and $\Gamma_{e}\left(t\right)=\alpha \text{exp}\left[-{\left(t-t_{2}\right)}^{2}/T^{2}\right]$, which show the peak value α at $t=t_{1}$ and $t=t_{2}$, respectively, and have a width 2T in the time domain. The adiabatic condition can be expressed as $\left|d\theta \left(t\right)/dt\right|\ll \sqrt{\Gamma_{e}{\left(t\right)}^{2}+\Gamma_{f}{\left(t\right)}^{2}}$ with $\theta \left(t\right)=\text{arctan}[\Gamma_{e}\left(t\right)/\Gamma_{f}\left(t\right)]$. This condition is, in principle, feasible due to the greatly suppressed decoherence of this braided giant-atom pair.

If the above Λ−V model is extended to a multiple-atom version where an array of such atomic dimers (with an alternating arrangement of Λ- and V-type atoms) is considered to form a 1D chain, and if each atom is only braided with its two nearest neighbors but is separated from all the other atoms [27], as shown in Fig. 1c, then a 1D *diamond lattice* consisting of the atomic upper states (e.g., $|e_{1}\rangle$, $|e_{2}\rangle$, and $|f_{2}\rangle$) can be realized as shown in Fig. 1d.

**Fig. 2:**
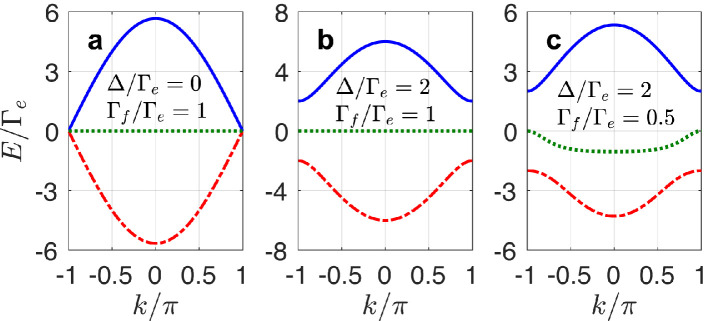
Band structures of the effective diamond lattice with (a) $\Delta /\Gamma_{e}=0$ and $\Gamma_{f}/\Gamma_{e}=1$, (b) $\Delta /\Gamma_{e}=2$ and $\Gamma_{f}/\Gamma_{e}=1$, and (c) $\Delta /\Gamma_{e}=2$ and $\Gamma_{f}/\Gamma_{e}=0.5$.

One intriguing feature of the diamond lattice in Fig. 1d is the existence of a *flat band* [38], i.e., a completely dispersionless energy band that allows for compact localized states [39]. More specifically, the dynamical equations of such a lattice can be given by:

(21) $${\dot{\tilde{a}}}_{m}=-2i\left[\Gamma_{e}({\tilde{b}}_{m}+{\tilde{b}}_{m-1})+\Gamma_{f}({\tilde{c}}_{m}+{\tilde{c}}_{m-1})\right],$$

(22) $${\dot{\tilde{b}}}_{m}=-2i\Gamma_{e}({\tilde{a}}_{m}+{\tilde{a}}_{m+1}),$$

(23) $${\dot{\tilde{c}}}_{m}=-2i\Gamma_{f}({\tilde{a}}_{m}+{\tilde{a}}_{m+1}),$$

where we have rewritten the atomic excitation amplitudes as $c_{e,2m-1}\to {\tilde{a}}_{m}$, $c_{e,2m}\to {\tilde{b}}_{m}$, and $c_{f,2m}\to {\tilde{c}}_{m}$ and assumed $\text{mod}\left(\phi ,2\pi \right)=\text{mod}\left(\phi^{\prime },2\pi \right)=\pi /2$. By using the plane-wave solutions ${\tilde{a}}_{m}\left(t\right),{\tilde{b}}_{m}\left(t\right),{\tilde{c}}_{m}\left(t\right)\propto \text{exp}\left(ikm-iEt\right)$, the eigenvalues of the effective diamond lattice can be obtained as

(24) $$E_{0}=0,\;E_{\pm }=\pm 2\sqrt{2\left(\Gamma_{e}^{2}+\Gamma_{f}^{2}\right)\left(1+\cos k\right)},$$

which show a flat band between two symmetric dispersive ones. Note that in this case, there is no band gap between these bands, as shown in Fig. 2a.

To open band gaps, one can introduce small frequency mismatching to either the Λ-type or the V-type atom so that the transition frequencies of two atoms are not exactly matched. In fact, this is a common situation in circuit QED since it is challenging to construct exactly matched Λ- and V-type atoms (although one can also simulate the three-level structures by using two two-level atoms). Fig. 2b and c show the *gapped* band structures of the diamond lattice in this case (here we assume $\omega_{e,2m}-\omega_{g,2m}=\omega_{e}+\Delta$ and $\omega_{f,2m}-\omega_{g,2m}=\omega_{f}-\Delta$ with Δ the small frequency mismatching; for more details see Sec. II in the supplemental material). As expected, the frequency mismatching opens gaps between these bands, with which the associated compact localized states can be robust against certain types of disorders [40]. Note that a complete flat band disappears if $\Gamma_{e}\neq \Gamma_{f}$. As shown in Fig. 2c, although the band gaps persist in this case, the middle band is nearly dispersionless around the middle and edges of the Brillouin zone but becomes dispersive elsewhere.

### 2.2 Double-Λ model

**Fig. 3:**
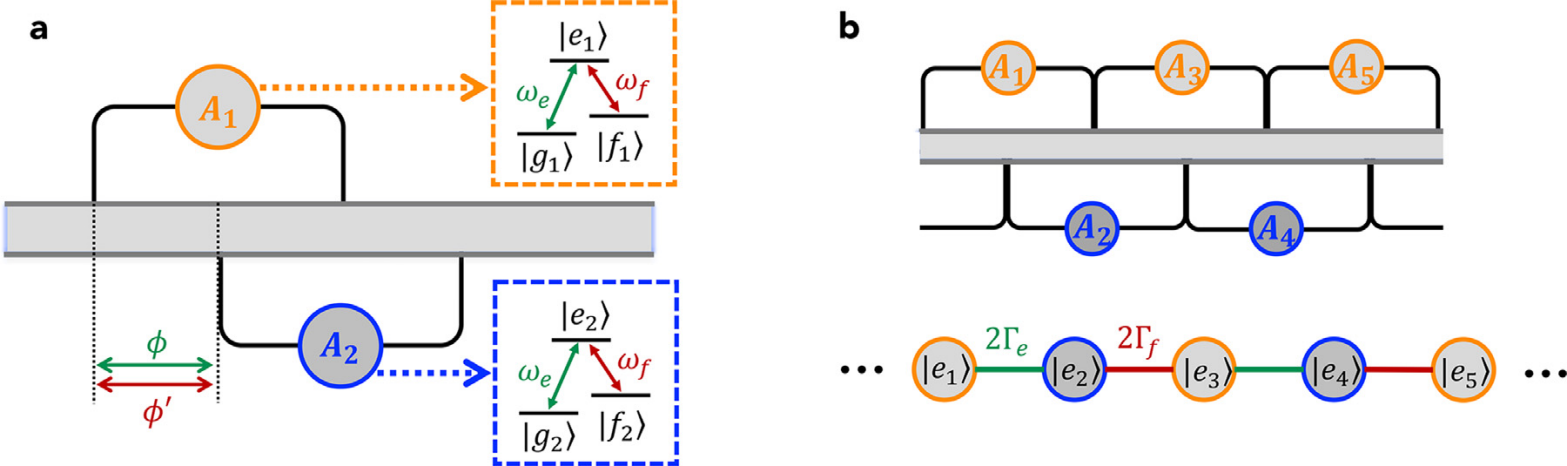
**(a) Schematic diagram of the double-**Λ**model**. The two Λ-type giant atoms are coupled to a common 1D waveguide with an equally-spaced braided structure. (b) Effective SSH lattice based on an array of the double-Λ models.

In this section, we turn to consider two identical Λ-type atoms coupled to the waveguide with the equally-spaced braided structure. As shown in Fig. 3a, the Hamiltonian of such a double-Λ model can be written as $H^{\prime }=H_{\text{w}}+H_{A_{1}}+H_{A_{2}}^{\prime }+V_{\text{w}1}+V_{\text{w}2}^{\prime }$, which is obtained by replacing $H_{A_{2}}$ and $V_{\text{w}2}$ in Eq. 1 by $H_{A_{2}}^{\prime }$ and $V_{\text{w}2}^{\prime }$, respectively, with

(25) $$H_{A_{2}}^{\prime }=\omega_{e}|e_{2}\rangle \langle e_{2}|+\Delta_{ef}|f_{2}\rangle \langle f_{2}|,$$

(26) $$V_{\text{w}2}^{\prime }=\int_{-\infty }^{+\infty }dk\left(e^{ikd}+e^{3ikd}\right)\left(g_{e}|e_{2}\rangle \langle g_{2}|+g_{f}|e_{2}\rangle \langle f_{2}|\right)a_{k}+\text{H.c.}.$$

If $A_{1}$ is initially prepared in state $|e_{1}\rangle$, there are *two* possible single-excitation states depending on which lower state $A_{2}$ is initially in: if $A_{2}$ is prepared in state $|g_{2}\rangle$ (we refer to it as “case A” hereafter), the state at time t is given by

(27) $$\begin{array}{ccc} {|\psi \left(t\right)\rangle }_{A} & = & \left[c_{e,1}\left(t\right)|e_{1}g_{2}\rangle +c_{e,2}\left(t\right)|g_{1}e_{2}\rangle \right]e^{-i\omega_{e}t}|0\rangle \\ & & +\;\int_{-\infty }^{+\infty }dk[\sum_{j=1,2}c_{k,j}^{\prime }\left(t\right)|f_{j}\rangle \langle g_{j}|e^{-i\Delta_{ef}t}\\ & & +\;c_{k,12}\left(t\right)]a_{k}^{†}e^{-i\omega_{k}t}|g_{1}g_{2}\rangle |0\rangle , \end{array}$$

whereas if $A_{2}$ is prepared in state $|f_{2}\rangle$ (we refer to it as “case B” hereafter), the state becomes

(28) $$\begin{array}{ccc} {|\psi \left(t\right)\rangle }_{B} & = & \{\left[c_{e,1}\left(t\right)|e_{1}f_{2}\rangle +c_{e,2}\left(t\right)|f_{1}e_{2}\rangle \right]e^{-i\omega_{e}t}\\ & & +\;\int_{-\infty }^{+\infty }dk[\sum_{j=1,2}c_{k,j}\left(t\right)|g_{j}\rangle \langle f_{j}|+c_{k,12}^{\prime }\left(t\right)\\ & & \times e^{-i\Delta_{ef}t}]a_{k}^{†}e^{-i\omega_{k}t}|f_{1}f_{2}\rangle \}e^{-i\Delta_{ef}t}|0\rangle . \end{array}$$

Here $c_{k,j}\left(t\right)$ [$c_{k,j}^{\prime }\left(t\right)$] is the time-dependent probability amplitude of atom $A_{j}$ in state |g〉 (|f〉) and the other one in state |f〉 (|g〉), while $c_{k,12}\left(t\right)$ [$c_{k,12}^{\prime }\left(t\right)$] is the time-dependent probability amplitude of both atoms in state |g〉 (|f〉). Note that the two atoms cannot be in state |f〉 (|g〉) simultaneously in case A (case B) during the time evolution.

By solving the time-dependent Schrödinger equation and following a similar calculation procedure as in the previous section, one can finally obtain:

(29) $$\begin{array}{ccc} {\dot{c}}_{e,1}\left(t\right) & = & -\left(\Gamma_{e}+\Gamma_{f}\right)c_{e,1}\left(t\right)-\left(\Gamma_{e}e^{2i\phi }+\Gamma_{f}e^{2i\phi^{\prime }}\right)D_{e,1}^{\left(2\right)}\\ & & -\;\frac{\Gamma_{e}}{2}\left[3e^{i\phi }D_{e,2}^{\left(1\right)}+e^{3i\phi }D_{e,2}^{\left(3\right)}\right], \end{array}$$

(30) $$\begin{array}{ccc} {\dot{c}}_{e,2}\left(t\right) & = & -\left(\Gamma_{e}+\Gamma_{f}\right)c_{e,2}\left(t\right)-\left(\Gamma_{e}e^{2i\phi }+\Gamma_{f}e^{2i\phi^{\prime }}\right)D_{e,2}^{\left(2\right)}\\ & & -\;\frac{\Gamma_{e}}{2}\left[3e^{i\phi }D_{e,1}^{\left(1\right)}+e^{3i\phi }D_{e,1}^{\left(3\right)}\right] \end{array}$$

in case A, and obtain:

(31) $$\begin{array}{ccc} {\dot{c}}_{e,1}\left(t\right) & = & -\left(\Gamma_{e}+\Gamma_{f}\right)c_{e,1}\left(t\right)-\left(\Gamma_{e}e^{2i\phi }+\Gamma_{f}e^{2i\phi^{\prime }}\right)D_{e,1}^{\left(2\right)}\\ & & -\;\frac{\Gamma_{f}}{2}\left[3e^{i\phi^{\prime }}D_{e,2}^{\left(1\right)}+e^{3i\phi^{\prime }}D_{e,2}^{\left(3\right)}\right], \end{array}$$

(32) $$\begin{array}{ccc} {\dot{c}}_{e,2}\left(t\right) & = & -\left(\Gamma_{e}+\Gamma_{f}\right)c_{e,2}\left(t\right)-\left(\Gamma_{e}e^{2i\phi }+\Gamma_{f}e^{2i\phi^{\prime }}\right)D_{e,2}^{\left(2\right)}\\ & & -\;\frac{\Gamma_{f}}{2}\left[3e^{i\phi^{\prime }}D_{e,1}^{\left(1\right)}+e^{3i\phi^{\prime }}D_{e,1}^{\left(3\right)}\right] \end{array}$$

in case B. Once again, in the Markovian regime of $\{\Gamma_{e}\tau ,\Gamma_{f}\tau \}\ll 1$ and if $\text{mod}\left(\phi ,2\pi \right)=\text{mod}\left(\phi^{\prime },2\pi \right)=\pi /2$ (i.e., $n=n^{\prime }=1$), the above equations can be simplified to

(33) $${\dot{c}}_{e,1}\left(t\right)=-2i\Gamma_{\beta }c_{e,2}\left(t\right),$$

(34) $${\dot{c}}_{e,2}\left(t\right)=-2i\Gamma_{\beta }c_{e,1}\left(t\right),$$

where β=e,f for cases A and B, respectively. Clearly, the two Λ-type atoms exhibit a decoherence-free interaction (i.e., the two atomic excited states exchange excitation without decaying into the waveguide), with *the effective coupling strength depending on which state* $A_{2}$ *is initially prepared in*. This intriguing feature allows us to implement the SSH model based on the atomic excited states. As shown in Fig. 3b, this can be accomplished by using an array of such double-Λ models, where the atoms are initially prepared in states |g〉 and |f〉 *alternately*.

**Fig. 4:**
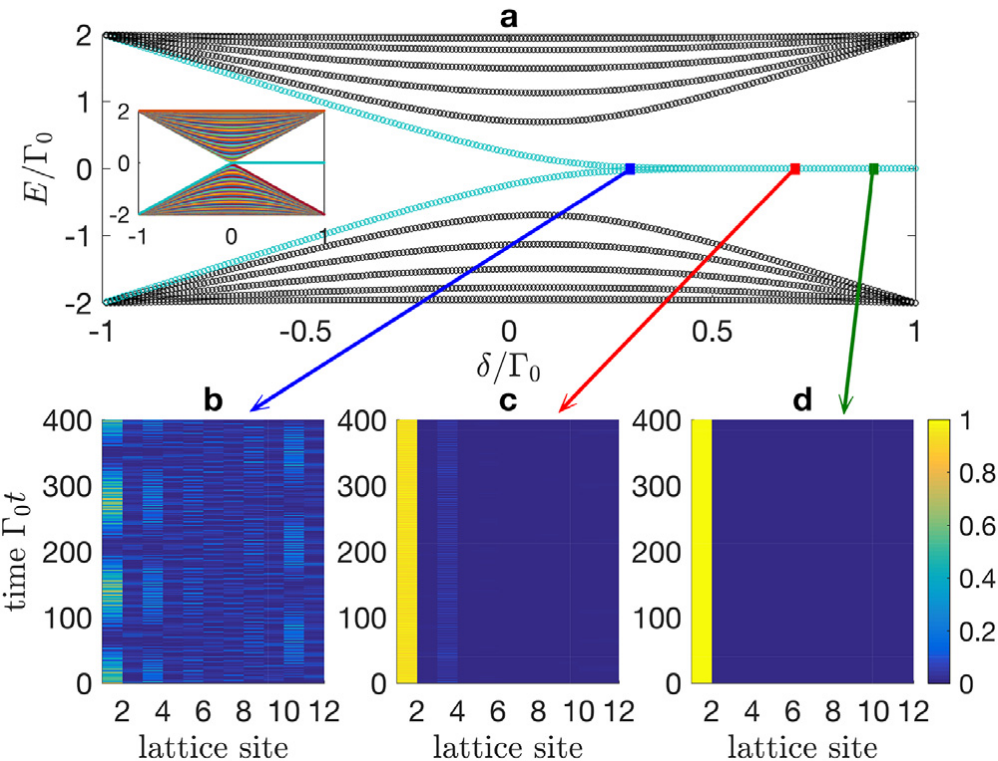
**(a) Energy spectrum of an effective SSH model with**M=6. The inset depicts the energy spectrum with M=50. (b)–(d) Time evolutions of the initial single-site excitation $c_{e,m}\left(t=0\right)=\delta_{m,1}$ for (b) $\delta \Gamma /\Gamma_{0}=0.3$, (c) $\delta \Gamma /\Gamma_{0}=0.7$, and (d) $\delta \Gamma /\Gamma_{0}=0.9$. We assume M=6 in panels (b)–(c). Other parameters are $\Gamma_{e}=\left(\Gamma_{0}-\delta \Gamma \right)/2$ and $\Gamma_{f}=\left(\Gamma_{0}+\delta \Gamma \right)/2$.

One of the most important features of the SSH model is the existence of topologically protected edge states which are localized at the two ends of the lattice (under open boundary conditions) [41]. We plot in Fig. 4a the real-space energy spectrum of the effective SSH model, which can be described by the Hamiltonian:

(35) $$H_{\text{SSH}}=\sum_{m=1}^{M}2\Gamma_{e}\left({\hat{a}}_{m}^{†}{\hat{b}}_{m}+\text{H.c.}\right)+\sum_{m=1}^{M-1}2\Gamma_{f}\left({\hat{b}}_{m}^{†}{\hat{a}}_{m+1}+\text{H.c.}\right)$$

with $2\Gamma_{e}=\Gamma_{0}-\delta \Gamma$ and $2\Gamma_{f}=\Gamma_{0}+\delta \Gamma$ describing the intracell and intercell couplings, respectively. In Eq. 35 we have defined ${\hat{a}}_{m}$ (${\hat{b}}_{m}$) as the annihilation operator of the effective lattice site $|e_{2m-1}\rangle$ ($|e_{2m}\rangle$) in the single-excitation subspace. Fig. 4a shows that a pair of zero-energy edge states appear in the middle band gap under open boundary conditions and when δΓ is positive. Such a topological phase transition tends to be ideal (showing an explicit phase transition point δΓ=0) in the thermodynamic limit M→+∞, as shown in the inset of Fig. 4a. These zero-energy states are protected by the chiral symmetry [42] of the lattice and thus robust against certain types of disorders.

We also examine in Fig. 4b–d the time evolutions of the initial single-site excitation $c_{e,m}\left(t=0\right)=\delta_{m,1}$ (i.e., the leftmost atom is initially prepared in state |e〉) for different values of $\delta \Gamma \in [-\Gamma_{0},\Gamma_{0}]$. For a large (positive) δΓ, it shows that the excitation maintains localized in the leftmost lattice site with no transfer along the lattice. As δΓ decreases, the excitation may partially leak to the nearest-neighbor lattice site, which can be understood from the edge state with a weaker localization strength in this case. Such a localization feature disappears, however, when δΓ becomes small enough. In this case, the excitation spreads along the lattice and is reflected back and forth by the lattice boundaries.

In contrast to the simulation scheme of SSH model based on the chiral bound states of two-level giant atoms [34], the present scheme works in a very different regime where the waveguide does not possess designed band gaps. Moreover, compared with the scheme in Ref. [35], where the SSH model is simulated based on the staggered braided structures (i.e., braided structures with designed unequal coupling separations) of two-level giant atoms, our effective SSH model is closely related to the initial states of the atoms and thus is *reconfigurable*. For instance, our model can reduce to a 1D tight-binding chain if the atoms are reset to the same lower state. Furthermore, one can also create an SSH heterostructure [43] by simply reversing the staggered atomic initial states within a chosen spatial range and engineer topologically protected interface states.

### 2.3 Double-V model

**Fig. 5:**
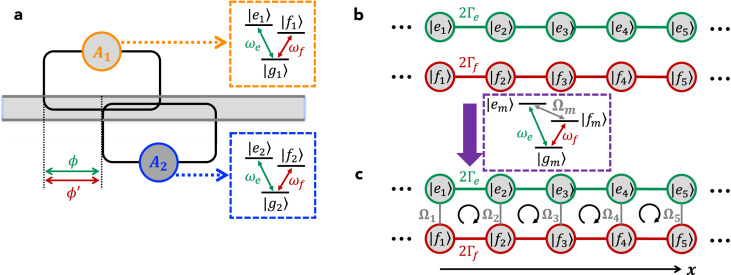
(a) Schematic diagram of the double- V model. The two V-type giant atoms are coupled to a common 1D waveguide with an equally-spaced braided structure. (b) Two independent effective 1D tight-binding lattices based on an array of the double-V models. (c) Effective ladder lattice with the help of external coherent fields.

Finally, we study another atomic dimer model where a pair of identical V-type atoms are coupled to the waveguide with the equally-spaced braided structure. As shown in Fig. 5a, the Hamiltonian of such a double-V model can be written as $H^{\prime \prime }=H_{\text{w}}+H_{A_{1}}^{\prime }+H_{A_{2}}+V_{\text{w}1}^{\prime }+V_{\text{w}2}$, which is obtained by replacing $H_{A_{1}}$ and $V_{\text{w}1}$ in Eq. 1 by $H_{A_{1}}^{\prime }$ and $V_{\text{w}1}^{\prime }$, respectively, with

(36) $$H_{A_{1}}^{\prime }=\omega_{e}|e_{1}\rangle \langle e_{1}|+\omega_{f}|f_{1}\rangle \langle f_{1}|,$$

(37) $$V_{\text{w}1}^{\prime }=\int_{-\infty }^{+\infty }dk\left(e^{ikd}+e^{3ikd}\right)\left(g_{e}|e_{1}\rangle \langle g_{1}|+g_{f}|f_{1}\rangle \langle g_{1}|\right)a_{k}+\text{H.c.}.$$

In contrast to the case in Section 2.2, here there are two possible single-excitation states depending on which *upper state* of $A_{1}$ (i.e., $|e_{1}\rangle$ or $|f_{1}\rangle$) is initially occupied. More specifically, if $A_{1}$ is initially in state $|e_{1}\rangle$ (we refer to it as “case C” hereafter), the state of the model at time t can be written as

(38) $$\begin{array}{ccc} {|\psi \left(t\right)\rangle }_{C} & = & \{\left[c_{e,1}\left(t\right)|e_{1}g_{2}\rangle +c_{e,2}\left(t\right)|g_{1}e_{2}\rangle \right]e^{-i\omega_{e}t}\\ & & +\;\int_{-\infty }^{+\infty }dkc_{k}\left(t\right)a_{k}^{†}e^{-i\omega_{k}t}|g_{1}g_{2}\rangle \}|0\rangle , \end{array}$$

whereas if $A_{1}$ is initially prepared in state $|f_{1}\rangle$ (we refer to it as “case D” hereafter), the state becomes

(39) $$\begin{array}{ccc} {|\psi \left(t\right)\rangle }_{D} & = & \{\left[c_{f,1}\left(t\right)|f_{1}g_{2}\rangle +c_{f,2}\left(t\right)|g_{1}f_{2}\rangle \right]e^{-i\omega_{f}t}\\ & & +\;\int_{-\infty }^{+\infty }dkc_{k}\left(t\right)a_{k}^{†}e^{-i\omega_{k}t}|g_{1}g_{2}\rangle \}|0\rangle . \end{array}$$

Here $c_{e,j}\left(t\right)$ [$c_{f,j}\left(t\right)$] is the time-dependent probability amplitude of atom $A_{j}$ in state |e〉 (|f〉) and the other one in the ground state |g〉; $c_{k}\left(t\right)$ is the time-dependent probability amplitude of both atoms in the ground state and creating a photon with wave vector k in the waveguide.

Once again, by solving the Schrödinger equation and following the same calculation procedure as in the previous sections, we have

(40) $${\dot{c}}_{e,1}\left(t\right)=-\Gamma_{e}\left[c_{e,1}\left(t\right)+e^{2i\phi }D_{e,1}^{\left(2\right)}\right]-\frac{\Gamma_{e}}{2}\left[3e^{i\phi }D_{e,2}^{\left(1\right)}+e^{3i\phi }D_{e,2}^{\left(3\right)}\right],$$

(41) $${\dot{c}}_{e,2}\left(t\right)=-\Gamma_{e}\left[c_{e,2}\left(t\right)+e^{2i\phi }D_{e,2}^{\left(2\right)}\right]-\frac{\Gamma_{e}}{2}\left[3e^{i\phi }D_{e,1}^{\left(1\right)}+e^{3i\phi }D_{e,1}^{\left(3\right)}\right]$$

in case C, and have:

(42) $${\dot{c}}_{f,1}\left(t\right)=-\Gamma_{f}\left[c_{f,1}\left(t\right)+e^{2i\phi^{\prime }}D_{f,1}^{\left(2\right)}\right]-\frac{\Gamma_{f}}{2}\left[3e^{i\phi^{\prime }}D_{f,2}^{\left(1\right)}+e^{3i\phi^{\prime }}D_{f,2}^{\left(3\right)}\right],$$

(43) $${\dot{c}}_{f,2}\left(t\right)=-\Gamma_{f}\left[c_{f,2}\left(t\right)+e^{2i\phi^{\prime }}D_{f,2}^{\left(2\right)}\right]-\frac{\Gamma_{f}}{2}\left[3e^{i\phi^{\prime }}D_{f,1}^{\left(1\right)}+e^{3i\phi^{\prime }}D_{f,1}^{\left(3\right)}\right]$$

in case D. Under the decoherence-free condition, e.g., $\text{mod}\left(\phi ,2\pi \right)=\text{mod}\left(\phi^{\prime },2\pi \right)=\pi /2$ ($n=n^{\prime }=1$), and in the Markovian regime of $\{\Gamma_{e}\tau ,\Gamma_{f}\tau \}\ll 1$, we have the simplified dynamical equations:

(44) $${\dot{c}}_{\beta ,1}\left(t\right)=-2i\Gamma_{\beta }c_{\beta ,2}\left(t\right),$$

(45) $${\dot{c}}_{\beta ,2}\left(t\right)=-2i\Gamma_{\beta }c_{\beta ,1}\left(t\right),$$

where β=e,f for cases C and D, respectively. Clearly, in both cases, the two V-type giant atoms behave just like a pair of coupled two-level systems [27], [28], with the effective coupling strength determined by the initial state. This implies that an array of such double-V models mimics two *independent* 1D tight-binding chains, as shown in Fig. 5b.

More interestingly, it is also possible to simulate a quasi-1D *ladder lattice* by introducing external coherent fields to drive the transitions between the two upper states $|e_{m}\rangle$ and $|f_{m}\rangle$, as shown in Fig. 5c. This is justified for three-level superconducting artificial atoms, where cyclic transition structures are allowed if the atoms are operated away from the optimal points [44], [45]. Note that the cyclic structure based on an external field does not break the single-excitation assumption [24].

Such a ladder lattice can exhibit nontrivial dispersion relations if a synthetic gauge field is introduced to each square plaquette [46]. This can be achieved by introducing external fields with a *phase gradient* along the x direction, i.e., $\Omega_{m}=\Omega \text{exp}\left(2im\phi \right)$, as shown in Fig. 5c. By performing the gauge transform $|e_{m}\rangle \to |e_{m}\rangle \text{exp}\left(im\phi \right)$ and $|f_{m}\rangle \to |f_{m}\rangle \text{exp}\left(-im\phi \right)$, the effective ladder lattice can be described by the Hamiltonian:

(46) $$H_{\text{ladder}}=-\sum_{m}\left(\Omega {\hat{a}}_{m}^{†}{\hat{b}}_{m}+2\Gamma_{e}e^{i\phi }{\hat{a}}_{m+1}^{†}{\hat{a}}_{m}+2\Gamma_{f}e^{-i\phi }{\hat{b}}_{m+1}^{†}{\hat{b}}_{m}+\text{H.c.}\right),$$

where ${\hat{a}}_{m}$ (${\hat{b}}_{m}$) now annihilates an excitation at the effective lattice site $|e_{m}\rangle$ ($|f_{m}\rangle$). Eq. 46 shows that each square plaquette of the ladder lattice is threaded by a synthetic gauge flux Φ=2ϕ. By using again the plane-wave solutions ${\hat{a}}_{m},\;{\hat{b}}_{m}\propto \text{exp}\left(ikm-iEt\right)$ and assuming $2\Gamma_{e}=2\Gamma_{f}=\Gamma$ for simplicity, the dispersion relation of the ladder lattice can be given by

(47) $$E_{\pm }=-2\Gamma \left[\cos\phi \cos k\mp \sqrt{\sin^{2}\phi \sin^{2}k+{\left(\frac{\Omega }{2\Gamma }\right)}^{2}}\right].$$

It is clear from Eq. 47 that the synthetic gauge field can significantly modify the dispersion relation of the lattice, leading to, e.g., the spin-momentum locking effect [46]. Moreover, one can define the average spin:

(48) $${\langle \sigma_{z}\rangle }_{k,\pm }=\mp \left[\cos^{2}\left(\frac{\theta_{k}}{2}\right)-\sin^{2}\left(\frac{\theta_{k}}{2}\right)\right]$$

for the energy bands $E_{\pm }$, respectively, with $\theta_{k}=\text{arctan}\left[\Omega /\left(2\Gamma \sin\phi \sin k\right)\right]$. The Bloch modes of the lattice prefer to occupy the sublattice consisting of states $|e_{m}\rangle$ ($|f_{m}\rangle$) if the corresponding average spin is positive (negative).

**Fig. 6:**
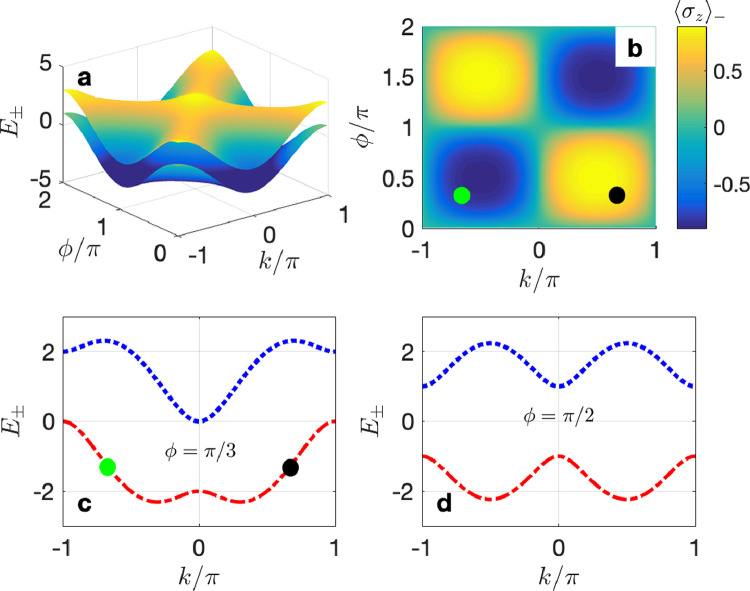
(a) Energy spectrum of the ladder lattice versus k and ϕ. (b) Average spin of the lower energy band versus k and ϕ. (c) and (d) Energy band profiles versus k for (c) ϕ=π/3 and (d) ϕ=π/2. The filled circles in panels (b) and (c) with the same color correspond to the same parameters. In this figure we assume Ω=Γ=1.

We plot in Fig. 6 the energy bands of the ladder lattice and the average spin of the lower band to illustrate the spin-momentum locking effect mentioned above. One can find that the energy bands are closely related to the synthetic gauge flux, which allows for simulating the spin-orbital coupling [46]. As shown in Fig. 6b–d, the “spin-up” (${\langle \sigma_{z}\rangle }_{k}>0$) and “spin-down” (${\langle \sigma_{k}\rangle }_{k}<0$) modes can propagate toward opposite directions along different sublattices for appropriate values of ϕ. For example, as shown by the green and black filled circles in Fig. 6b and c, the Bloch modes with opposite wave vectors k≈±0.665π have opposite group velocities and thus propagate toward opposite directions along different sublattices. In this case, it is possible to observe chiral spontaneous emission of a quantum emitter if it is coupled to this lattice and resonant with one of the energy bands [46].

## 3 Conclusion

In conclusion, we have demonstrated a series of 1D and quasi-1D synthetic photonic lattices with protected tunnelings and nontrivial band structures. By designing pairs of Λ- and V-type three-level giant atoms, as summarized in Table 1, it is possible to create different decoherence-free level structures, which can further serve as unit cells of, e.g., diamond, SSH, and ladder lattices. More advanced lattice models can be expected by considering more types of multi-level giant atoms.

**Table 1:** **Synthetic lattices with different giant-atom models**.

Atomic pair	Effective level configuration	Atomic array
Λ−V model	Λ-type configuration	Diamond lattice
Double-Λ model	Two-level configuration	SSH model
	(with initial-state-dependent strength)	
Double-V model	Two-level configuration	1D tight-binding chains (without external fields)
	(with initial-state-dependent strength)	Ladder lattice (with external fields)

Although giant-atom systems are theoretically achievable with waveguide QED platforms based on cold atoms or various solid-state quantum emitters, it is more convenient to implement our proposals with superconducting quantum circuits where one can tune the level structure of an artificial three-level atom on demand [44], [45] and the atom-field interactions can be engineered by using various tunable couplers [37], [47], [48]. The braided coupling structure can be easily realized by using a meandering (e.g., S-type) superconducting transmission line such that the first (second) coupling point of $A_{2}$ can be located between (outside) the two coupling points of $A_{1}$ [27], [28]. In fact, a similar giant-atom system has recently been demonstrated in experiments where two frequency-tunable transmon qubits, i.e., two-level artificial atoms, are coupled to a meandering 50−Ω coplanar waveguide in the braided manner [28]. Another advantage of implementing giant-atom systems with superconducting quantum circuits is that the atom-field coupling points can be well controlled. For cold-atom platforms, however, one should consider the small oscillations of the atoms trapped in harmonic optical potentials [49]. Such oscillations may result in time-varying phase accumulations and, thereby, smeared giant-atom interference effects. Note that decoherence-free interactions between giant atoms are allowed even if the braided coupling points are not equally spaced (although the interaction strengths would be modified in this case) [27]. In practice, it is challenging to precisely control the positions of all the coupling points. This amounts to introducing tunneling disorders to the synthetic lattices. However, this is a common challenge for most existing simulation schemes. Moreover, some topologically nontrivial features, such as the edge states of the SSH model, are robust against this type of disorder since it does not break the chiral symmetry of the lattice model.

Several advantages of our proposals are summarized as follows. First, *next-nearest-neighbor couplings* are greatly inhibited due to the braided coupling structure [27]. This prevents undesired long-range interactions from smearing the synthetic lattice [50], which however remains a challenge for many other platforms [51], [52], [53]. For example, for Zigzag coupled waveguide arrays, next-nearest-neighbor couplings can be up to 30% of the nearest-neighbor ones if the intra-layer waveguide separation is twice the distance between the layers [54]. Second, for circuit QED implementations, other decay channels of the artificial atoms (such as their intrinsic dissipations into the non-guided modes of the environment) can be made very small. Therefore, using our schemes, one can construct a series of large-scale photonic lattices with, however, weak enough decoherence. Third, one can readily turn on and off the interaction between two chosen adjacent lattice sites (amounts to creating a boundary there) by tuning the corresponding atomic transitions in and out of resonance with each other. Moreover, some of the schemes proposed in this paper (e.g., those based on the double-Λ and double-V models) are *reconfigurable*. One can simulate different lattice models by changing the initial states of the atoms without reconstructing the architecture of the system. Last but not the least, although we have focused on decoherence-free interactions in this paper, controllable dissipations (into the waveguide) can be introduced to the atoms (i.e., the lattice sites) by tuning the coupling separations of the braided structure. This provides the possibility to simulate non-Hermitian photonic lattices with structured loss [55].

## CRediT authorship contribution statement

**Lei Du:** Investigation, Methodology, Writing – original draft. **Yan Zhang:** Funding acquisition, Supervision, Writing – review & editing. **Xin Wang:** Methodology, Validation. **Yong Li:** Funding acquisition, Supervision, Writing – review & editing. **Yu-xi Liu:** Supervision, Project administration, Writing – review & editing.

## Declaration of competing interest

The authors declare that they have no conflicts of interest in this work.

## Appendix A Supplementary materials

Supplementary Data S1 Supplementary Raw Research Data. This is open data under the CC BY license http://creativecommons.org/licenses/by/4.0/ Supplementary Data S1
