# Supplementary material for: Synthetic photonic lattices based on three-level giant-atom arrays
Source: Fundam Res. 2024 May 9;6(1):162–9. doi: 10.1016/j.fmre.2024.03.029 (PMC12869755; doi:10.1016/j.fmre.2024.03.029)
Supplement: Supplementary Data S1 — Supplementary Raw Research Data. This is open data under the CC BY license http://creativecommons.org/licenses/by/4.0/ [file mmc1.pdf]

Supplemental material for "Synthetic photonic lattices based on three-level giant-atom arrays"

Lei Du,[1,2] Yan Zhang,[3,*] Xin Wang,[4] Yong Li,[1,†] and Yu-xi Liu[5]

[1] *Center for Theoretical Physics & School of Physics and Optoelectronic Engineering, Hainan University, Haikou 570228, China*
[2] *Department of Microtechnology and Nanoscience, Chalmers University of Technology, 412 96 Gothenburg, Sweden*
[3] *School of Physics and Center for Quantum Sciences, Northeast Normal University, Changchun 130024, China*
[4] *Institute of Theoretical Physics, School of Physics, Xi'an Jiaotong University, Xi'an 710049, China*
[5] *School of Integrated Circuits, Tsinghua University, Beijing 100084, China*

I. DERIVATION PROCESS OF EQS. (15)-(17)

Recalling the Λ-V model as shown in Fig. 1(a), one can obtain the dynamical equations Eqs. (8)-(12) by solving the time-dependent Schrödinger equation in the single-excitation subspace. By substituting the formal solutions Eqs. (13) and (14) of the field amplitudes into Eqs. (8)–(10), we have

$$\begin{aligned}\dot{c}_{e,1}(t) &= -\int_0^t dt'\int_{-\infty}^{+\infty} dk\Big\{\left(2+e^{2ikd}+e^{-2ikd}\right)\left[g_e^2 e^{-i\Delta_e(t-t')}+g_f^2 e^{-i\Delta_f(t-t')}\right]c_{e,1}(t')\\ &\quad+\left(e^{ikd}+2e^{-ikd}+e^{-3ikd}\right)\left[g_e^2 c_{e,2}(t')e^{-i\Delta_e(t-t')}+g_f^2 c_{f,2}(t')e^{-i\Delta_f(t-t')}\right]\Big\}, \qquad \text{(S1)}\\ \dot{c}_{e,2}(t) &= -g_e^2\int_0^t dt'\int_{-\infty}^{+\infty} dk\Big[\left(2+e^{2ikd}+e^{-2ikd}\right)c_{e,2}(t')+\left(2e^{ikd}+e^{-ikd}+e^{3ikd}\right)c_{e,1}(t')\Big]e^{-i\Delta_e(t-t')}, \qquad \text{(S2)}\\ \dot{c}_{f,2}(t) &= -g_f^2\int_0^t dt'\int_{-\infty}^{+\infty} dk\Big[\left(2+e^{2ikd}+e^{-2ikd}\right)c_{f,2}(t')+\left(2e^{ikd}+e^{-ikd}+e^{3ikd}\right)c_{e,1}(t')\Big]e^{-i\Delta_f(t-t')}. \qquad \text{(S3)}\end{aligned}$$

By changing the integration variable, i.e., $\int_{-\infty}^{+\infty}F(k)dk \rightarrow \int_{-\infty}^{+\infty}[F(k)+F(-k)]d\Delta_e/v_g$ with $F(k)$ being a function of k, and using the definition $\int_{-\infty}^{+\infty}\exp(-ikx)dk = 2\pi\delta(x)$ of the δ function, Eqs. (S1)–(S3) become

$$\begin{aligned}\dot{c}_{e,1}(t) &= -\frac{2\pi}{v_g}\int_0^t dt'\Big\{\left[4\delta(t-t')+2e^{2i\phi}\delta(t-t'-2\tau)+2e^{-2i\phi}\delta(t-t'+2\tau)\right]\left[g_e^2+g_f^2 e^{-i\Delta_{ef}(t-t')}\right]c_{e,1}(t')\\ &\quad+\left[3e^{i\phi}\delta(t-t'-\tau)+3e^{-i\phi}\delta(t-t'+\tau)+e^{3i\phi}\delta(t-t'-3\tau)+e^{-3i\phi}\delta(t-t'+3\tau)\right]\\ &\quad\times\left[g_e^2 c_{e,2}(t')+g_f^2 c_{f,2}(t')e^{-i\Delta_{ef}(t-t')}\right]\Big\}, \qquad \text{(S4)}\\ \dot{c}_{e,2}(t) &= -\frac{2\pi g_e^2}{v_g}\int_0^t dt'\Big\{\left[4\delta(t-t')+2e^{2i\phi}\delta(t-t'-2\tau)+e^{-2i\phi}\delta(t-t'+2\tau)\right]c_{e,2}(t')\\ &\quad+\left[3e^{i\phi}\delta(t-t'-\tau)+3e^{-i\phi}\delta(t-t'+\tau)+e^{3i\phi}\delta(t-t'-3\tau)+e^{-3i\phi}\delta(t-t'+3\tau)\right]c_{e,1}(t')\Big\}, \qquad \text{(S5)}\\ \dot{c}_{f,2}(t) &= -\frac{2\pi g_f^2}{v_g}\int_0^t dt' e^{-i\Delta_{ef}(t-t')}\Big\{\left[4\delta(t-t')+2e^{2i\phi}\delta(t-t'-2\tau)+2e^{-2i\phi}\delta(t-t'+2\tau)\right]c_{f,2}(t')\\ &\quad+\left[3e^{i\phi}\delta(t-t'-\tau)+3e^{-i\phi}\delta(t-t'+\tau)+e^{3i\phi}\delta(t-t'-3\tau)+e^{-3i\phi}\delta(t-t'+3\tau)\right]c_{e,1}(t')\Big\}, \qquad \text{(S6)}\end{aligned}$$

where $\phi = \omega_e\tau = \omega_e d/v_g$ is the phase accumulation of a photon with frequency ω_e traveling between two adjacent coupling points and τ describes the time delay of this propagation process. By using the property $\int_{-\infty}^{+\infty}\delta(k-k')F(k)dk = F(k')$ of the δ function, Eqs. (S4)–(S6) can be further simplified to Eqs. (15)–(17) in the main text.

* zhangy345@nenu.edu.cn
† yongli@hainanu.edu.cn

II. SYNTHETIC DIAMOND LATTICES WITH BAND GAPS

In Eqs. (21)-(23), we have assumed exactly matched transition frequencies $\omega_{e,1}-\omega_{g,1}=\omega_{e,2}-\omega_{g,2}\equiv\omega_e$ and $\omega_{e,1}-\omega_{f,1}=\omega_{f,2}-\omega_{g,2}\equiv\omega_f$ for the two atoms, which is however a challenge in experiments. If we consider a slightly off-resonant case, where $\omega_{e,2}-\omega_{g,2}=\omega_e+\Delta$ and $\omega_{f,2}-\omega_{g,2}=\omega_f-\Delta$ with Δ a small frequency mismatch, the dynamical equations of the synthetic diamond lattice can be immediately written as

$$\dot{\tilde{a}}_m = -2i\left[\Gamma_e\left(\tilde{b}_m+\tilde{b}_{m-1}\right)+\Gamma_f\left(\tilde{c}_m+\tilde{c}_{m-1}\right)\right], \tag{S7}$$

$$\dot{\tilde{b}}_m = -i\Delta\tilde{b}_m-2i\Gamma_e\left(\tilde{a}_m+\tilde{a}_{m+1}\right), \tag{S8}$$

$$\dot{\tilde{c}}_m = i\Delta\tilde{c}_m-2i\Gamma_f\left(\tilde{a}_m+\tilde{a}_{m+1}\right), \tag{S9}$$

and the corresponding eigenvalues become

$$E_1 = \left[-\frac{q}{2}+\sqrt{\left(\frac{q}{2}\right)^2+\left(\frac{p}{3}\right)^3}\right]^{1/3}+\left[-\frac{q}{2}-\sqrt{\left(\frac{q}{2}\right)^2+\left(\frac{p}{3}\right)^3}\right]^{1/3}, \tag{S10}$$

$$E_2 = \alpha\left[-\frac{q}{2}+\sqrt{\left(\frac{q}{2}\right)^2+\left(\frac{p}{3}\right)^3}\right]^{1/3}+\alpha^*\left[-\frac{q}{2}-\sqrt{\left(\frac{q}{2}\right)^2+\left(\frac{p}{3}\right)^3}\right]^{1/3}, \tag{S11}$$

$$E_3 = \alpha^*\left[-\frac{q}{2}+\sqrt{\left(\frac{q}{2}\right)^2+\left(\frac{p}{3}\right)^3}\right]^{1/3}+\alpha\left[-\frac{q}{2}-\sqrt{\left(\frac{q}{2}\right)^2+\left(\frac{p}{3}\right)^3}\right]^{1/3} \tag{S12}$$

where $p=-\left[8\left(1+\cos k\right)\left(\Gamma_e^2+\Gamma_f^2\right)+\Delta^2\right]$, $q=-8\Delta\left(1+\cos k\right)\left(\Gamma_e^2-\Gamma_f^2\right)$, and $\alpha=(\alpha^*)^*=\left(-1+\sqrt{3}i\right)/2$.
